# Reference Percentiles and Changes over Time for Total Thyroxine in Preterm Infants: A Retrospective Cohort Study

**DOI:** 10.3390/diagnostics10070475

**Published:** 2020-07-13

**Authors:** Claudia M. Flores-Robles, Ernesto Roldan-Valadez, Nayeli Martínez-Cruz, Lidia Arce-Sánchez, Ana L. Priego-Zurita, Guadalupe Estrada-Gutierrez, Enrique Reyes-Muñoz

**Affiliations:** 1 Department of Endocrinology, Instituto Nacional de Perinatología “Isidro Espinosa de los Reyes”, Montes Urales 800, 11000 Mexico City, Mexico; cmontsefr@gmail.com (C.M.F.-R.); nayemc_21@hotmail.com (N.M.-C.); li_arce@yahoo.com.mx (L.A.-S.); 2 Directorate of Research, Hospital General de Mexico “Dr. Eduardo Liceaga”, Dr. Balmis #148, 06720 Mexico City, Mexico; ernest.roldan@usa.net; 3 Department of Radiology, I.M. Sechenov First Moscow State Medical University (Sechenov University), 119146 Moscow, Russia; 4 Pediatric Endocrinology, Hospital Infantil de México “Federico Gómez”, Dr. Marquez #162, 06720 Mexico City, Mexico; nalypriego@gmail.com; 5 Research Division, Instituto Nacional de Perinatología, “Isidro Espinosa de los Reyes”, Montes Urales 800, 11000 Mexico City, Mexico; gpestrad@gmail.com; 6 Coordination of Gynecological and Perinatal Endocrinology, Instituto Nacional de Perinatología “Isidro Espinosa de los Reyes”, Montes Urales 800, 11000 Mexico City, Mexico

**Keywords:** thyroxine, newborn screening, congenital hypothyroidism, hypothyroxinemia, central Hypothyroidism, preterm infants, dried blood spot

## Abstract

Hypothyroxinemia of prematurity increases the rate of false-positive results in total thyroxine (tT4)-based screening programs for congenital hypothyroidism. The use of specific cutoff values for preterm infants has been proposed, but data on tT4 reference ranges in this population are limited. The primary aim was to establish reference percentiles for tT4 in dried blood spots among Mexican preterm infants. Secondary aims included a comparison of the change of tT4 concentrations over time according to gestational age and to discuss its impact on tT4-based screening programs. This was a retrospective cohort study; 1561 preterm infants were included. Percentile 10th for tT4 concentration at 24–27, 28–30, 31–34, and 35–36 weeks of gestational age, measured in the first week of life was: 47.6, 56.6, 82.3, and 117.1 nmol/L, respectively. tT4 concentrations were compared in three different time points: first week of life, 2–3 weeks of life, and term-corrected gestational age (38 weeks of gestation), progressively increased in infants below 30 weeks, remained stable in infants from 31 to 34 weeks, and decreased in late preterm newborns (35–36 weeks). This study suggests that preterm infants may require the use of lower tT4 cutoff values in newborn screening.

## 1. Introduction

Newborn screening (NS) for congenital hypothyroidism (CH) is one of the most useful tools for preventing severe mental retardation [1]. Most NS programs worldwide employ a primary thyroid-stimulating hormone (TSH)-based methodology that detects only primary congenital hypothyroidism (PCH), responsible for the majority of CH cases [2]. PCH occurs due to defective thyroid gland development (thyroid dysgenesis) or hormone biosynthetic defect (dyshormonogenesis) [3]. The incidence of CH has increased recently, especially in preterm newborns, with an overall incidence of 1:1714, and up to 1:62 among preterm infants born before 32 weeks of gestation [4].

Central congenital hypothyroidism (CCH) is less common than PCH, with a reported incidence of 1:16,000 to 1:21,600 [3,5]. This incidence is comparable to other conditions included in NS programs such as phenylketonuria or galactosemia [3,6]. CCH results from inappropriate thyroid hormone production caused by defective stimulation of a normal thyroid gland by the thyroid-stimulating hormone (TSH) [3]. CCH manifests with normal or subnormal TSH concentration despite subnormal thyroid hormone concentration [3,5]. Only the programs in which total thyroxine (tT4) or free thyroxine (fT4) are measured simultaneously with TSH or in a stepwise manner with an initial tT4 measurement are capable of detecting CCH [2].

One of the principal disadvantages of tT4-based programs is the high false-positive rate, caused mainly by hypothyroxinemia of prematurity and thyroxine-binding globulin (TBG) deficiency [5]. It is well documented that during the first weeks of life, preterm infants have lower concentration of thyroid hormones compared to term newborns, and that tT4 concentration correlate positively with gestational age and inversely with the severity of their illness [5,7]. Lower T4 concentrations in preterm newborns are one of the causes of the lower efficacy of tT4-based NS programs in this subgroup of infants [8].

There is controversy around the world over which is the best newborn screening strategy for CH in preterm infants. Several studies have shown that serial TSH testing improves detection of CH [4,9], but this strategy fails to detect CCH. Some authors have suggested using specific cutoff values for this population, but there is no consensus on which TSH or tT4 cutoff values for preterm infants should be used [4,10,11].

Using the same tT4 cutoffs for preterm and term neonates increases the recall rate in NS in preterm infants and could lead to misdiagnosis or overdiagnosis of CH [8]. Data on tT4 reference ranges on NS in preterm neonates is scarce, therefore, the primary aim of this study was to determine reference percentiles for tT4 in Mexican preterm infants. As secondary aims, we analyzed how tT4 concentration changes over time in preterm infants according to their gestational age and discuss the implications that this could have on tT4-based screening programs for preterm infants.

## 2. Materials and Methods

### 2.1. Study Design and Patients

This is a retrospective cohort study performed in a tertiary care hospital in Mexico City. All preterm infants born before 37 weeks of gestation from January 2018 to December 2019 were included. The following infants were excluded: infants whose first NS was done before 24 h, infants diagnosed with primary or central CH, neonatal hyperthyroidism or persistent hyperthyrotropinemia, and infants who did not complete the institutional screening strategy or in whom confirmatory tests to rule out CH could not be performed for any reason. The study was performed according to the principles of the Declaration of Helsinki and was approved by the Ethic and Research Committees of the Instituto Nacional de Perinatología Isidro Espinoza de los Reyes; register number 2020-1-12, 7 May 2020.

### 2.2. Screening Methods

All newborn infants were screened for CH according to the institutional NS program, serial testing of simultaneous tT4 and TSH were conducted. The first specimen was collected between two and five days of life, the second specimen was collected in all preterm infants between the 2nd and 3rd weeks of life (2 weeks after the first screening) and the third and last specimen was collected at term-corrected gestational age (38 weeks of gestation). This last specimen was obtained only in preterm infants born before 34 weeks of gestation. The institutional screening program cutoff value for TSH is 5 mIU/L, lower than the national cutoff of 10 mIU/L [12], and the tT4 cutoff is the 5th percentile from our population of term and preterm infants (90 nmol/L in serum or 45 nmol/L in whole blood). Confirmatory tests (serum thyroid profile) were requested in the following cases: (a) TSH >5 mIU/L at any sample or (b) tT4 below the 5th percentile in the first and second specimens. PCH was defined as persistent TSH >10 mIU/L regardless of tT4 concentration and CCH as TSH <10 mIU/L in addition of a fT4 below 10.3 pmol/L associated with midline malformations, hypoglycemia, hypopituitarism, central nervous system abnormalities, or persistently low fT4 after exclusion of hypothyroxinemia of prematurity or other differential diagnoses.

### 2.3. Laboratory Methods

tT4 concentration was measured by solid-phase, time-resolved fluoroimmunoassay from dried blood spots (DBS) by using Perkin Elmer’s AutoDELFIA platform (Waltham, MA, USA). Intra and interassay coefficients of variation (CV) were 5.9 and 7.9%, respectively, for a tT4 of 13.5 μg/dL, with a functional sensitivity of 1.5 μg/dL. The TSH and tT4 measurements were then converted to estimated serum concentration with the following conversion factor: a T4 blood level of 0.5 μg/dL = a serum level of 1 μg/dL, a TSH blood concentration of 1 mIU/mL = a serum concentration of 2 mIU/L according to the manufacturer’s recommendations (assuming 50–55% hematocrit).

The samples were taken in the following settings: Neonatal Intensive Care Unit (NICU, Level III of care), Neonatal Intermediate Care Unit (IMC, Level II of care), Neonatal Minimal Care Unit (MCU, Level 1 of Care), and obstetrics wards or ambulatory [13].

### 2.4. Data Collection

Infant’s demographics, NS results, and confirmatory test results were obtained from the electronic medical record of each infant. All serial tT4 values of the study cohort were included in the analysis. tT4 values obtained between days 2 and 7 of life were recorded as tT4 values at first screening (week 1), tT4 values obtained between days 7 and 21 as tT4 values at second screening (week 2–3), and tT4 values obtained after these time periods as tT4 values at third screening (term-corrected gestational age or 38 weeks’ gestation). Collected specimens from other time points were excluded. Reference percentiles for preterm infants were presented in 4 groups according to differences in their thyroid hormone physiology [7,14]; Group 1, 24 to 27 weeks; Group 2, 28 to 30 weeks; Group 3, 31 to 34 weeks; and Group 4, 35 to 36 weeks.

### 2.5. Statistical Analysis

Results were expressed either as means and standard deviations (SD) for continual variables or median and interquartile ranges when the distribution was not normal; the numerical count and the percentage for categorical variables were used. We studied the normal distribution of tT4 concentrations in the four study groups and found non-normal distribution in the following gestational age groups (28 to 30 weeks, 31 to 34 groups, and 35 to 36 weeks). tT4 percentiles were reported for the 4 gestational age groups in three-time points (week1, weeks 2 to 3, and term-corrected gestational age). The following percentiles were calculated: 5th, 10th, 25th, 50th, 75th, 90th, and 95th.

#### Comparisons of tT4 and TSH between Gestational Age Groups and Screening Times

We compared tT4 and TSH concentration between gestational age groups in the three different screening time points with the Kruskal–Wallis test. To search for a linear trend during the 3 screening times for each gestational age group we performed a Friedman test as the non-parametric equivalent of the repeated measures ANOVA test; for Group 4 (35–36 weeks) we performed a Wilcoxon signed-rank test, as this group only had 2 screening times. All tests were considered significant at an alpha level of 0.05. All statistical analysis was performed using the IBM^®^ SPSS^®^ Statistics software (version 24, IBM Corporation; Armonk, NY, USA).

## 3. Results

### 3.1. Newborn Screening Results

5969 infants were screened at the institute between January 2018 and December 2019, of which 1614 (27%) were born before 37 weeks. Screening tests were positive in 81 preterm infants (recall rate 5%), 40 had TSH above the institutional cutoff in any of the three serial samples, 39 had tT4 values in their first and second screening below the 5th percentile, and two infants screened positive for both parameters, tT4 and TSH. The recall rate for a positive tT4 screening (2 serial tT4 below the 5th percentile) was inversely proportional to the gestational age, being 14% in Group 1 (24–27 weeks), 11.6% in Group 2 (28–30 weeks), 3.3% in Group 3 (31–34 weeks), and only 0.2% in Group 4 (35–36 weeks).

Eight cases of congenital hypothyroidism were confirmed during the study period, four PCH, two cases of CCH (one with congenital panhypopituitarism, and 1 with septo-optic dysplasia) and two with transient CCH associated with maternal Graves’ disease. The false-positive rate was 4.5%, the positive predictive value was 9.8% and the total incidence of CH in our population was 1 in 269 preterm births (1:403 for PCH and 1:807 CCH, excluding infants with known transient CCH).

### 3.2. Infants Included for tT4 Percentiles

The final cohort included 1561 preterm infants after excluding 53 infants on the basis of exclusion criteria: 2 infants whose first NBS was done before 24 h, 11 infants diagnosed with primary or central CH, neonatal hyperthyroidism or persistent hyperthyrotropinemia, and 40 infants without complete screening strategies or without confirmatory tests to rule out CH (it was not possible because of death, palliative care or any other reason). Table 1 shows the demographic characteristics of the infants included.

#### Serial Screening Samples for tT4 Percentiles

1549 (99.2%) of infants completed the first screening, 1430 (91.6%) completed the second screening, and from those who were born before 34 weeks of gestation, only 571 (81.3%) completed the third screening due to being lost to follow-up. Samples were obtained in the following settings: First screening, 15.5% in NICU, 32.2% in ICU, 23.6% in MCU, and 28.7% in obstetrics wards or ambulatory; Second screening, 11.7% in NICU, 31.6% in ICU, 2.2% in MCU, and 54.5% in obstetrics wards or ambulatory; Third screening, 5.4% in NICU, 40.5% in ICU, 1.7% in MCU, and 52.4% in obstetrics wards or ambulatory.

### 3.3. Primary Outcome: tT4 Percentiles

Table 2 shows the 5th to 95th percentiles for tT4 in the three screening times: first screening (week 1), second screening (week 2 to 3), and third screening (term-corrected gestational age).

### 3.4. Comparisons of tT4 between Gestational Age Groups

**Figure 1:**
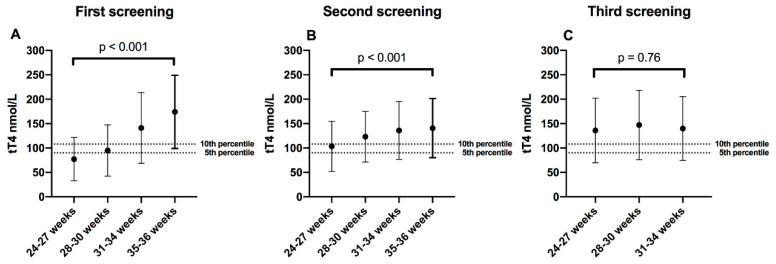
Medians of total thyroxine (tT4) in serial screening; (**A**) First screening = week 1; (**B**) Second screening = week 2–3; (**C**) Third screening = 38 corrected gestational weeks. The dotted lines were placed above the 5th and 10th percentile, which are the most-used tT4 screening cutoff points worldwide (in our population it equals to 90 nmol/L and 108 nmol/L). Error bars represent the 5th and 95th percentile of each gestational group. tT4 comparisons between groups were made with the Kruskal–Wallis test.

tT4 concentration was higher at higher gestational age in first and second screening, tT4 medians (IQR) in first screening were as follows: Group 1 (24–27 weeks) = 75.9 (61.7–90.1) nmol/L, Group 2 (28–30 weeks) = 87.5 (73.3–113.2) nmol/L; Group 3 (31–34 weeks) = 132.5 (106.8–160.9) nmol/L, and Group 4 (35–36 weeks) = 164.7 (137.1–194.3) nmol/L; *p* < 0.001. At second screening, Group 1 = 104.2 (90.1–124.8) nmol/L, Group 2 = 114.5 (95.2–139) nmol/L, Group 3 = 126.1 (108.1–150.6) nmol/L, and Group 4 = 130 (110.6–154.4) nmol/L; *p* < 0.001. In the third screening (term-corrected gestational age or 38 gestational weeks), tT4 concentration was similar in all gestational age groups: Group 1 = 127.4 (108.1–162.1) nmol/L, Group 2 = 133.8 (110.6–158.3) nmol/L and Group 3, = 130 (105.5–160.9) μg/dL; *p* = 0.76; Figure 1.

### 3.5. Comparisons of tT4 between Screening Times

**Figure 2:**
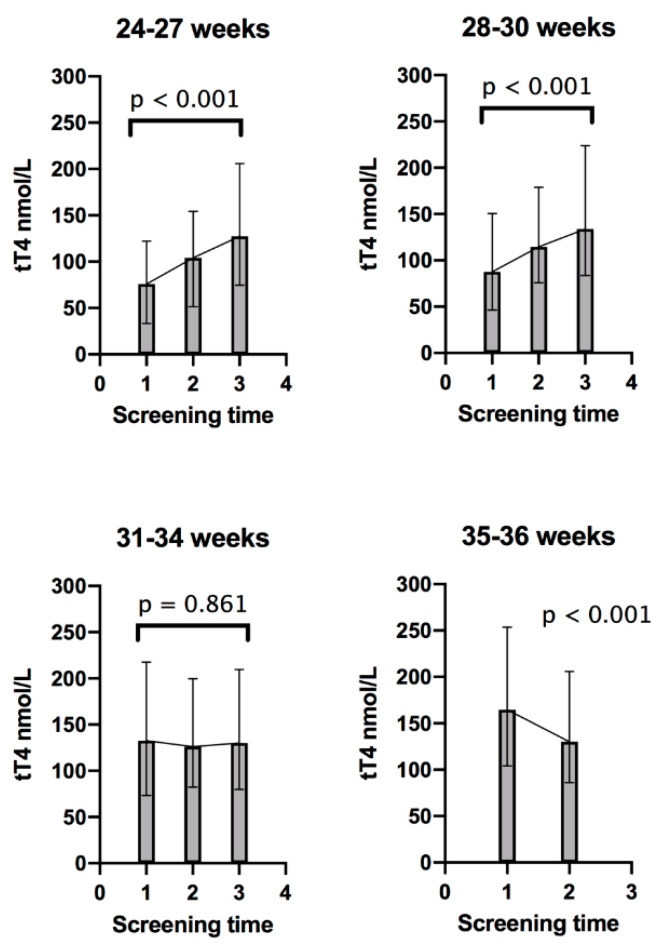
Trends of total thyroxine (tT4) in each gestational age group; 1 = First screening (week 1); 2 = Second screening (week 2–3); and 3 = Third screening (38 corrected gestational weeks). Error bars represent the 5th and 95th percentile for each screening time. tT4 comparisons between sample times for each group were made with the Friedman test, except for Group 4, which was compared with Wilcoxon signed-rank test.

tT4 concentrations progressively increased in the three screening time points in Group 1, infants born at 24 to 27 weeks, *p* < 0.001, and Group 2, infants born at 28 to 30 weeks, *p* < 0.001. However, in Group 3, infants born at 31 to 34 weeks, tT4 concentration was similar in the three screening times, *p =* 0.861. In Group 4, 35 to 36 weeks, tT4 concentration decreased in the second screening compared to the first screening, *p* < 0.001; Figure 2.

### 3.6. Comparisons of TSH between Screening Times

**Figure 3:**
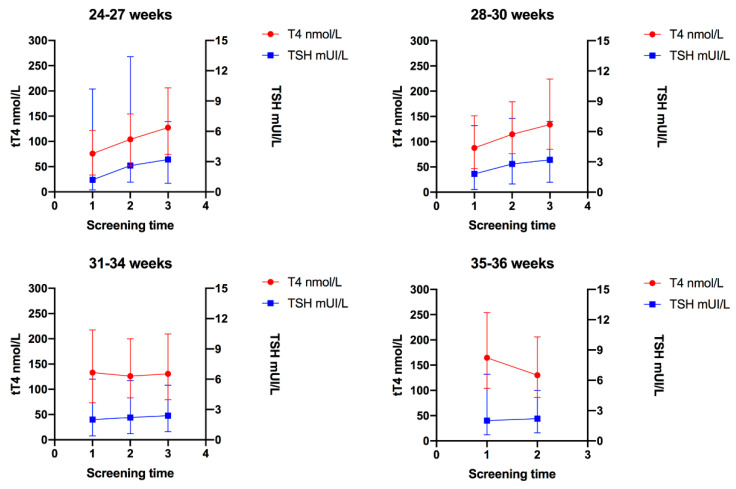
Trends of total thyroxine (tT4) and thyroid-stimulating hormone (TSH) in each gestational age group; 1 = First screening (week 1); 2 = Second screening (week 2–3); and 3 = Third screening (38 corrected gestational weeks). Error bars represent the 5th and 95th percentile for T4 and TSH in each screening time.

TSH concentration progressively increased in the three screening time points in Group 1 to 3, in Group 4, TSH concentration was similar in the two screening time points. The medians of TSH were as follows: Group 1 (24–27 weeks), first screening = 1.2 mIU/L, second screening = 2.6 mIU/L, third screening = 3.2 mIU/L, *p* < 0.001; Group 2 (28–30 weeks), first screening = 1.8 mIU/L, second screening = 2.8 mIU/L, third screening = 3.2 mIU/L, *p* < 0.001; Group 3 (31–34 weeks), first screening = 2 mIU/mL, second screening = 2.2 mIU/mL, third screening = 2.4 mIU/mL, *p* < 0.001; Group 4 (35–36 weeks), first screening = 2 mUI/L, second screening = 2.2 mIU/L, *p* = 0.086. Figure 3 shows the change in TSH concentrations relative to the change in T4.

## 4. Discussion

This study allowed us to obtain tT4 reference ranges in DBS for Mexican preterm infants in three different time points (first week of life: 2–5 days of life, 2–3 postnatal weeks, and term-corrected gestational age); to our knowledge, this is the first study in which serial tT4 values in DBS are described in preterm newborns.

Few studies [15,16,17,18] have evaluated the course of tT4 beyond four weeks of life in preterm infants, most of them [16,17,18] have included a relatively small sample and have measured tT4 concentration on serum and not in DBS. These studies [15,16,17,18] reported that in preterm infants below 30 weeks, tT4 concentration declines in the first weeks of life and returns to normal concentration between four and eight weeks of life, which is consistent with our results.

In agreement with previous studies, we found that tT4 concentration increases at higher gestational ages [7,14,19], however, this association was present only in screening samples obtained in the first four weeks of life, as tT4 concentration was similar in all gestational age groups in samples obtained at 38 weeks of corrected age.

Recently, Hijman et al. [20] determined reference values for tT4 in DBS for term and preterm infants born in Zurich, in samples obtained during the first week of life. In agreement with our results, they found a higher tT4 concentration at higher gestational ages. In Zurich, a TSH-based strategy is used, thus, in order to obtain tT4 values, stored DBS samples had to be used and tT4 results from infants with abnormal TSH (as only infants with a TSH value above cutoff had a direct tT4 measurement); these circumstances, coupled with the differences in our populations, could account for the lower tT4 concentration reported by them [20].

This study showed that in preterm infants below 30 weeks, tT4 concentration increased progressively; in preterm infants from 31 to 34 weeks, tT4 concentrations were stable at three screening timepoints; and finally, in late preterm newborns (35–36 weeks) tT4 concentration decreased at the second screening (2 to 3 weeks of age) timepoint compared to the first screening (first week of life) timepoint. These results differ from those reported by Williams et al. [19], who found that the normal tT4 postnatal surge, which occurs in term infants during the first week of life, is attenuated in preterm infants at 31–34 weeks, absent at 28–30 weeks and reverted in 23–27 weeks. However, they measured tT4 in serum, not in DBS, and measured tT4 at four different postnatal time points (0, 7, 14, and 28 days of life) [15]. Late preterm infants in our cohort probably presented the normal TSH and tT4 surge reported previously in term infants during the first days of life, and then, the gradual decline in tT4 and T3 concentration after the first postnatal week [7,14,19].

When we analyzed the change over time in TSH concentrations in each gestational age group, we found that T4 medians increased progressively in premature infants born before 34 weeks and remained stable in infants born at 35 to 36 weeks. Although it is well known that term infants had a postnatal TSH surge in the first 24 h of life and that TSH concentrations then gradually decrease over time, it has been reported that this postnatal TSH surge may be abolished or attenuated in premature infants [14], which may explain our findings. Recently, Kaluarachchi et al. [11] obtained TSH reference ranges for preterm infants. They found that in extremely preterm infants (22 to 27 weeks), the median TSH increased from 2.1 mIU/mL at birth to 3.1 mIU/mL at the term-corrected gestational age, which was similar to our results, however, they found that the 95th TSH percentile was highest at birth and gradually declined until the 10th postnatal week of life. We also found that the 95th TSH percentile declined at term-corrected gestational age in all groups, except for Group 2.

Preterm infants represent a challenge for NS programs as they present higher false-positive and false-negative test results compared with term newborns, this has been attributed to factors such as the immaturity of the hypothalamic-pituitary-thyroid axis, reduced capacity for synthesis of thyroid hormones, presence of concomitant critical diseases, and the use of certain drugs (such as steroids, dopamine, and caffeine) [7,21]. Hypothyroxinemia of prematurity is a well-described condition affecting between 35 and 85% preterm infants and is a major cause of a high rate of false-positives on tT4-based screening programs [22]. Currently, there is no evidence that early detection or treatment of transient hypothyroxinemia improves neurological prognosis, morbidity or mortality of this population, therefore, its detection should not be an objective of NS programs [22,23,24].

There is worldwide controversy about which is the best screening strategy for CH, especially in preterm infants. Serial TSH testing has shown to improve detection of CH, however, this strategy is not capable of detecting CCH [9,21]. Korzeniewski et al. [25] evaluated the efficacy of four screening strategies used in Michigan between 1994 and 2010 and found that although tandem tT4 and TSH testing was associated with the highest detection rate of CH, the false-positive rate (FPR) was more than eight times greater than with a primary TSH method (FPR of 4.45% with tandem tT4 and TSH versus 0.54% with primary TSH and serial testing in infants weighing <1800 g). They estimated that an additional 297 false-positive determinations would be incurred for each additional case of CH detected.

Few studies have evaluated the effectiveness of a primary tT4-based strategy specifically in preterm infants, a recent study conducted in the USA showed that a tT4 screening strategy consisting of two serial tT4 samples, first at 24 h of life and second at 10–14 days (cutoff of tT4 <10th percentile in the first sample a and tT4 <0.5th percentile and/or TSH >30 mIU/mL in the second sample), showed poor efficacy in preterm infants with a birth weight lower than 1500 g hospitalized in an NICU unit. In this cohort, almost 40% of infants <1500 g had a positive screening test with a calculated sensitivity of 62.5%, a specificity of 79.1%, and a positive predictive value of only 8.1% [8]. In the present study, if the 10th percentile had been used as a cutoff point (2 serial tT4 below 10th percentile), instead of the institutional cutoff (5th percentile), the recall rate would have increased to 48.6% in Group 1, 29.4% in Group 2, 9.7% in Group 3, and 1.5% in Group 4, so almost half of the infants between 24 and 27 weeks would have required a confirmatory test.

Most NS programs with a simultaneous tT4 and TSH or tT4-based strategy uses the 5th or 10th percentile as the tT4 cutoff point [2,25,26]. Some authors have suggested the use of specific tT4 or TSH cutoffs in preterm infants with the objective of improving the efficacy of NS programs, however, there is limited evidence on which cutoffs are the most appropriate [10,11,27]. Defining appropriate cutoff values for this population is not a simple task, and achieving an adequate balance between minimizing the rate of false-positive results and maximizing the detection rate is complex. tT4 concentration between children with CCH, transient hypothyroxinemia of prematurity, and TBG deficiency can overlap, which makes this task even more difficult [5,26,28]. According to our results, we think than serial testing with tT4 could have a role in differentiating CCH from transient hypothyroxinemia.

In our cohort study, if we use tT4 cutoff from our total population of term and preterm infants in their first week of life, more than 70% of preterm infants of 24 to 27 weeks had a tT4 concentration below the 5th percentile (90 nmol/L in serum) and 85% below the 10th percentile (108 nmol/L in serum); among infants between 28 and 30 weeks, more than 50% had a tT4 concentration below the 5th percentile and more than 70% below the 10th percentile. To minimize recall and false-positive rates, our program has decided to request confirmatory thyroid profiles only in infants with two serial tT4 concentrations below the 5th percentile, even so, our recall rate in preterm infants continues to be high. It is also important to highlight that this strategy has allowed us to detect some cases of CCH in a timely manner (2 cases in the study period).

Including tT4 in NS programs increases the operating costs of them, however, the benefit of CCH detection outweighs these costs, as countries with a screening strategy capable of detecting CCH have shown a higher prevalence of this condition than previously reported [3,5,6], confirmatory testing methods for CCH are not as expensive as those required for other diseases included in screening programs, effective treatment is widely available and the consequences of late diagnosis are well known [1].

Limitations of this study include its retrospective design, although the data were collected prospectively by the institutional NS program. Another limitation is that we include a few tT4 measurements of infants of 24–27 gestational weeks (*n* = 36), as the frequency of births at this gestational age is not so high. Furthermore, in almost 20% of infants below 34 weeks, the third screening sample was not available due to loss to follow-up, many of these lost infants were discharged before reaching this gestational age and they did not come back for the last screening. It is possible that infants who were in a more critical situation and remained hospitalized were more likely to be included at this time point and this could decrease the tT4 concentrations reported. We decided to include sick neonates (as those requiring ventilatory support or pressor agents) because we wanted the reference ranges to reflect the real situation of these preterm infants.

The strengths of the present study include that we were able to report tT4 percentiles in preterm infants from three different postnatal time points (as our institute uses a simultaneous serial tT4 and TSH strategy), and tT4 values were obtained directly in DBS, not in stored DBS cards or serum samples, as one of our objectives was to obtain reference ranges for tT4 in preterm infants that could be useful to define screening cutoffs in the future.

To establish recommendations on which are the best cutoff points, multi-center studies with a higher number of preterm infants are required, as well as studies that directly compare the sensitivity and specificity of different cutoff points.

## 5. Conclusions

The results of this study suggest that preterm infants may require the use of lower DBS tT4 cutoff values according to gestational age at birth. tT4 concentrations increase progressively in infants below 30 weeks, remained stable in infants from 31 to 34 weeks, and decrease in late preterm newborns (35–36 weeks). To improve the effectiveness of screening strategies in preterm infants, each country should design their own protocols to define which tT4 cutoff values best optimize sensibility and specificity considering their health care system resources.

## Figures and Tables

**Table 1:** Baseline characteristics of preterm infant included in the cohort.

Characteristics		*n* = 1561
Gender, Male, *n* (%)		787 (50.4)
Gestational age, *n* (%)		
	24–27 weeks	37 (2.4)
	28–30 weeks	146 (9.4)
	31–34 weeks	519 (33.2)
	35–36 weeks	859 (55)
Birth weight, *n* (%)		
	<1000 g	95 (6.1)
	1001–1500 g	232 (14.9)
	1501–2000 g	379 (24.2)
	2001–2500 g	472 (30.2)
	>2500 g	384 (24.6)
Days of life at screening, median (IQR) *		
	First screening	3 (0)
	Second screening	18 (1)
	Third screening	41 (22)
Maternal history, *n* (%)		
	Thyroid disease	125 (8)
	Multiple pregnancies	390 (25)
Mode of delivery, *n* (%)		
	Vaginal	270 (17.3)
	Cesarean section	1291 (82.7)
Congenital malformations, *n* (%)		79 (5.1)

* IQR: Interquartile range.

**Table 2:** Reference percentiles for total thyroxine among Mexican preterm infants according to gestational age.

Gestational Age (Weeks)	*n*	5th	10th	25th	50th	75th	90th	95th	Units
First screening: week 1, *n* = 1549									
24–27	35	2.6	3.7	4.8	5.9	7.0	8.9	9.5	μg/dL
		33.4	47.6	61.7	75.9	90.1	114.5	122.2	nmol/L
28–30	146	3.6	4.4	5.7	6.8	8.8	10.4	11.7	μg/dL
		46.3	56.6	73.3	87.5	113.2	133.8	150.6	nmol/L
31–34	514	5.7	6.4	8.3	10.3	12.5	15.0	16.9	μg/dL
		73.3	82.3	106.8	132.5	160.9	193.1	217.5	nmol/L
35–36	854	8.1	9.1	10.7	12.8	15.1	17.6	19.7	μg/dL
		104.2	117.1	137.7	164.7	194.3	226.5	253.5	nmol/L
Second screening: week 2–3, *n* = 1430									
24–27	36	4.0	5.3	7.0	8.1	9.7	11.0	12.0	μg/dL
		51.4	68.2	90.1	104.2	124.8	141.5	154.4	nmol/L
28–30	143	5.9	6.4	7.4	8.9	10.8	12.6	13.9	μg/dL
		75.9	82.3	95.2	114.5	139	162.1	178.9	nmol/L
31–34	494	6.4	7.1	8.4	9.8	11.7	14.0	15.5	μg/dL
		82.3	91.4	108.1	126.1	150.6	180.2	199.5	nmol/L
35–36	757	6.7	7.3	8.6	10.1	12	14.2	16	μg/dL
		86.2	93.9	110.6	130	154.4	182.7	205.9	nmol/L
Third screening: Term-corrected gestational age (38 weeks of gestation), *n* = 571									
24–27	32	5.8	7.6	8.4	9.9	12.6	14.5	16.0	μg/dL
		74.6	97.8	108.1	127.4	162.1	186.6	205.9	nmol/L
28–30	117	6.5	7.0	8.6	10.4	12.3	15.1	17.4	μg/dL
		83.6	90.1	110.6	133.8	158.3	194.3	223.9	nmol/L
31–34	422	6.2	6.8	8.2	10.1	12.5	14.7	16.3	μg/dL
		79.8	87.5	105.5	130	160.9	189.2	209.7	nmol/L

Total thyroxine values represent estimated serum concentration with the following conversion factor: a blood level of 0.5 μg/dL = a serum level of 1 ug/dL. To obtain whole blood values, they must be divided by 2.
